# Syndromic (phenotypic) diarrhea in early infancy

**DOI:** 10.1186/1750-1172-3-6

**Published:** 2008-02-28

**Authors:** Olivier Goulet, Christine Vinson, Bertrand Roquelaure, Nicole Brousse, Christine Bodemer, Jean-Pierre Cézard

**Affiliations:** 1 Pediatric Gastroenterology-Hepatology and Nutrition, Reference Center for Rare Digestive Disease, Hôpital Necker-Enfants Malades/AP-HP, University of Paris 5 – René Descartes, France; 2 Pediatric Gastroenterology and Nutrition, Reference Center for Rare Digestive Disease, Hôpital Robert Debré/AP-HP, University of Paris 7, France; 3 Pediatric Gastroenterology and Nutrition, Hôpital de la Timone, University of Marseille, France; 4 Department of Pathology, Hôpital Necker-Enfants Malades/AP-HP, University of Paris 5 – René Descartes, France; 5 Pediatric Dermatology, Reference Center for Rare Dermatologic Disease, Hôpital Necker-Enfants Malades/AP-HP, University of Paris 5 – René Descartes, France

## Abstract

Syndromic diarrhea (SD), also known as phenotypic diarrhea (PD) or tricho-hepato-enteric syndrome (THE), is a congenital enteropathy presenting with early-onset of severe diarrhea requiring parenteral nutrition (PN). To date, no epidemiological data are available. The estimated prevalence is approximately 1/300,000–400,000 live births in Western Europe. Ethnic origin does not appear to be associated with SD. Infants are born small for gestational age and present with facial dysmorphism including prominent forehead and cheeks, broad nasal root and hypertelorism. Hairs are woolly, easily removed and poorly pigmented. Severe and persistent diarrhea starts within the first 6 months of life (≤ 1 month in most cases) and is accompanied by severe malabsorption leading to early and relentless protein energy malnutrition with failure to thrive. Liver disease affects about half of patients with extensive fibrosis or cirrhosis. There is currently no specific biochemical profile, though a functional T-cell immune deficiency with defective antibody production was reported. Microscopic analysis of the hair show twisted hair (pili torti), aniso- and poilkilotrichosis, and trichorrhexis nodosa. Histopathological analysis of small intestine biopsy shows non-specific villous atrophy with low or no mononuclear cell infiltration of the lamina propria, and no specific histological abnormalities involving the epithelium. The etiology remains unknown. The frequent association of the disorder with parental consanguinity and/or affected siblings suggests a genetic origin with an autosomal recessive mode of transmission. Early management consists of total PN. Some infants have a rather milder phenotype with partial PN dependency or require only enteral feeding. Prognosis of this syndrome is poor, but most patients now survive, and about half of the patients may be weaned from PN at adolescence, but experience failure to thrive and final short stature.

Syndromic diarrhea – Phenotypic diarrhea – Tricho-hepato-enteric syndrome – Intractable diarrhea of infancy with facial dysmorphism – Trichorrhexis nodosa and cirrhosis – Neonatal hemochromatosis phenotype with intractable diarrhea and hair abnormalities – Intractable infant diarrhea associated with phenotypic abnormalities and immune deficiency.

## Background

To date, several types of early onset intractable diarrhea of infancy (IDI) have been recognized [1-8]. Some of them involve primary epithelial abnormalities such as microvillous atrophy also called microvillous inclusion disease [5] and, more recently, intestinal epithelial dysplasia also called tufting enteropathy [6,7], while others are related to autoimmune disorders or complex syndromes involving mitochondrial disease or glycosylation proteins. The so-called "syndromic diarrhea" is an IDI syndrome associated with phenotypic abnormalities [9].

### Definition

Syndromic diarrhea (SD), also known as Phenotypic diarrhea (PD) or Tricho-hepato-enteric syndrome (THE), is a congenital enteropathy presenting with early-onset severe intractable diarrhea in infants born Small for Gestational Age (SGA) and associated with non-specific villous atrophy with low or no mononuclear cell infiltration of the lamina propria nor specific histological abnormalities involving the epithelium. The diarrhea is associated with facial dysmorphism, immune disorders and, in some patients, early onset of severe liver cirrhosis.

### History of the description

SD is a newly described clinicopathologic entity with intractable diarrhea in infants. Two cases have been reported by Stankler *et al. *as unexplained diarrhea and failure to thrive in two siblings with unusual facies and abnormal scalp hair shafts [10]. To date, the largest series involving 8 cases presenting a syndrome of intractable diarrhea associating phenotypic abnormalities and immune deficiency has been reported by Girault D *et al. *in 1994 [9]. Further case reports have confirmed the existence of the new entity [11-15]. Some cases reported by Girault D *et al*.,1994 presented with an early-onset severe cholestatic disease that rapidly progressed to cirrhosis and death [9]. A recent report (including two cases with severe liver disease) and the review of the published cases suggested that these patients have the same heterogeneous disease (inappropriately separated into different entities), suggesting that SD and THE are two sides of a now well recognized disease of unclear origin [15].

## Epidemiology

SD appears to be much less common than microvillous atrophy [5,7] or intestinal epithelial dysplasia [6,8]. Many cases are not yet recognized since the description of this disorder is recent. To date, no epidemiological data are available. However, the prevalence can be estimated at around 1/300,000–400,000 live births in Western Europe. The largest cohort of patients has been reported at the Necker-Enfants Malades Hospital in Paris, France [9]. The prevalence does not seem to differ significantly between ethnic groups. The disease seems to be more common in regions with a higher degree of consanguinity.

## Clinical description

**Figure 1:**
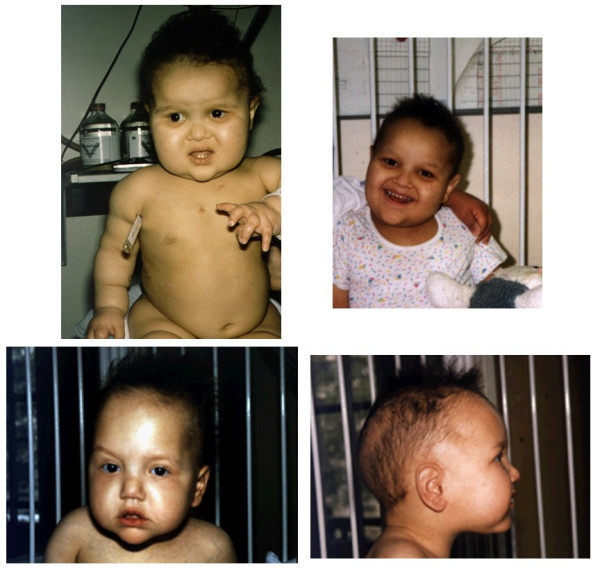
**Typical facial dysmorphism with prominent forehead and cheeks, broad nasal root and hypertelorism.** Abnormal hairs are woolly, easily removed and poorly pigmented.

**Figure 2:**
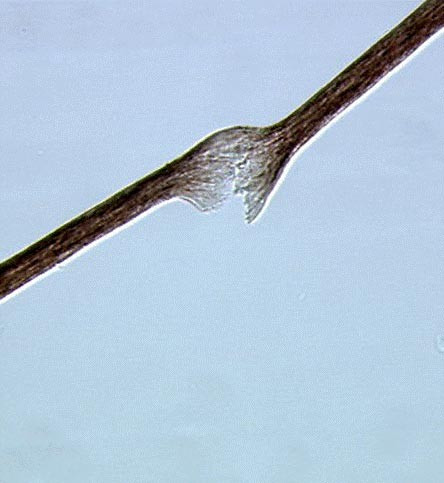
Microscopic analysis of the hair shaft showing trichorrhexis nodosa and longitudinal breaks.

The patients present with diarrhea starting within the first 6 months of life (≤ 1 month in most cases). Severe malabsorption leads to early and severe protein energy malnutrition with failure to thrive and patients require parenteral nutrition (PN). Diarrhea persists while on bowel rest on PN. All affected infants have several features in common [Additional file 1]. They are small for gestional age (<10° percentile) and have an abnormal phenotype. All have facial dysmorphism with prominent forehead and cheeks, broad nasal root and hypertelorism (Figure 1). Most children have difficulties with fine motor movements and are mentally retarded. They have a distinct hair abnormality: woolly hair that is easily removed and poorly pigmented even in children of Middle Eastern origin. Microscopic analysis of the hair shaft reveals non-specific abnormalities: twisted hair (pili torti), aniso- and poilkilotrichosis, trichorrhexis nodosa and longitudinal breaks (Figure 2), and trichothiodystrophy. Some cases were reported with trichorrhexis blastysis under scanning electron microscopy [10,11]. In the same cases, biochemical analysis of hairs revealed several anomalies of the amino acid pattern, including a low cystine content in the cases of trichothiodystrophy. There is currently no specific biochemical profile. Around half of the patients have liver disease [Additional file 1].

### Histological presentation

**Figure 3:**
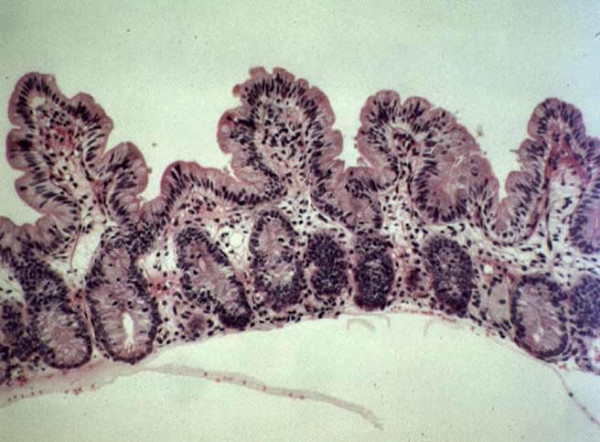
**Small intestine biopsy of a patient with syndromic diarrhea showing moderate villous atrophy with low degree of mononuclear cell infiltration in the lamina propria.** (Courtesy of Prof. Nicole Brousse, Hôpital Necker, Paris, France)

**Figure 4:**
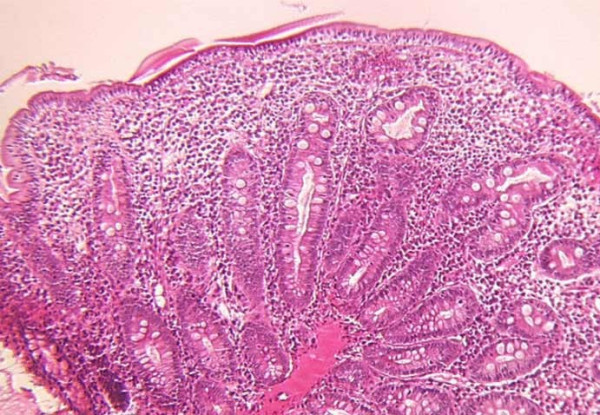
**Small intestine biopsy of a patient with syndromic diarrhoea showing severe villous atrophy with intense mononuclear cell infirltration in the lamina propria.** (Courtesy of Prof. Michel Peuchmaur, Hôpital Robert Debré, Paris, France)

From the reported cases in the literature, biopsies were performed during gastrointestinal endoscopy, at the time of referral to institutions or later at intervals depending on the therapeutic schedule. Biopsy specimens were stained with hematoxylin and eosin. Small intestine biopsies of the patients with SD show moderate (Figure 3) or severe villous atrophy with inconstant mononuclear cell infiltration of the lamina propria and absence of epithelial abnormalities (Figure 4). Histopathologically, there are no specific abnormalities. Few data using electron microscopy currently exist and, therefore, a precise description is not available. From our own experience (unpublished data) electron microscopy showed normal organization of the brush border, absence of anomalies of desmosomes and no remarkable picture suggesting ulstrastructural morphological changes. Extensive case reports and specimen collection should allow further studies to be performed.

In patients presenting with liver disease, pathological analysis of the liver usually shows macronodular cirrhosis with normal extra-hepatic ducts. Light microscopy examination shows extensive fibrosis or cirrhosis. Perl's staining shows iron depositions involving the hepatocytes and, to a lesser extent, the Kupfer cells. This aspect is consistent with neonatal hemochromatosis as suggested by Verloes *et al. *[11].

### Immune profile

Published profiles have been studied by performing a complete immunologic work-up, including analysis of the T- and B- cell populations, and mitogen (PHA, concavaline A, PWM), antigen (*Candida albicans*, tetanus toxoid) and allogenic cell-induced lymphocyte proliferation. Serum immunoglobulin levels and specific antibody titers to poliovirus, tetanus, *Diphteria toxoid *and *Bordetella pertussis *were also measured in most reports. Functional T-cell immune deficiency with defective antibody production was described in the original report [9]. Patients had defective antibody responses despite normal serum immunoglobulin levels, and defective antigen-specific skin tests despite positive proliferative responses *in vitro*. Further reports, in which extensive immunologic work-ups have been done, confirmed immune dysfunction. Several cases presented with monoclonal hyper IgA and/or hypo IgG [9,12]. The search for an abnormal immune profile and immune dysfunction should be part of the diagnosis work-up in the case of suspected SD.

## Etiopathogenesis

Among the congenital forms of hair dysplasia, tricorrhexis nodosa (TN) is very common and can be present in several pathologic conditions [16-19]. TN is the most common defect in the hair shaft, leading to hair breakage [20]. The primary abnormality is a focal loss of cuticles, causing fraying of the cortical fibers [21,22]. As emphasized by Landers *et al.*, TN may occur congenitally or can be acquired from chemical or physical trauma. Congenital TN has been associated with several syndromes including arginosuccinaciduria [23], citrullinemia [24,25], Menkes syndrome [26], Netherton disease [27,28], and syndromes associated with trichothiodystrophy hair shaft defect [29]. SD patients have defective antibody responses despite normal serum immunoglobulin levels, and defective antigen-specific skin tests despite positive proliferative responses *in vitro*. The cause of this functional immune deficiency and of this severe protracted diarrhea is unknown. The relationship between low birth weight, dysmorphism, severe diarrhea, hair shaft defect, immune deficiency and neonatal hemochromatosis-like liver disease is unclear. The coexistence of morphological, trichological and immunological abnormalities with early-onset intractable diarrhea disproportionate to the mucosal architectural abnormality (consistent with a primary enterocyte abnormality) suggests either mutations within several genes, inherited together by linkage disequilibrium, or, more probably, interference with a higher level of control, such as a patterning gene as seen in the Netherton syndrome which is an autosomal recessive congenital ichthyosis featuring chronic inflammation of the skin, hair anomalies, epidermal hyperplasia with an impaired epidermal barrier function, failure to thrive and atopic manifestations. The disease is caused by mutations in the *SPINK5 *gene encoding the serine proteinase inhibitor lympho-epithelial Kazal-type inhibitor [30,31]. The characteristic hair abnormalities may allow a more focused search for candidate mutations, as relatively few genes have been implicated in hair development.

### Mode of transmission

The frequent association of the disorder with parental consanguinity and/or affected siblings suggests a genetic origin with an autosomal recessive transmission [Additional file 1]. The gene involved in this congenital inherited disease has not yet been identified. Ethnic origin does not appear to be associated with the disease. Extensive case reports and specimen collection should allow future genetic studies to be performed.

## Diagnostic criteria

Diagnosis may be suspected early from the clinical presentation with the association of the following anomalies:

-intra-uterine growth retardation

-severe protracted diarrhea of early onset

-abnormal face with prominent forehead and cheeks

Most patients also have abnormal hair with tricorrhexis nodosa, immune deficiency, long-term growth failure and mental retardation in common. Liver disease is associated in about half of the patients and is variable in severity.

## Management and outcome

Early management consists of total parenteral nutrition (TPN) using central venous catheter in an experienced clinical setting. Patients usually have persistent diarrhea and, in enterally fed patients, malabsorption is severe. Its mechanisms are unknown, as villous atrophy is usually not as severe as the diarrhea is, and small bowel bacterial overgrowth or specific malabsorption have never been documented in these patients. Long-term PN is required for achieving growth even if catch up growth, in patients born SGA, cannot be achieved. Attempts at enteral feeding should be performed in all cases using semi-elemental diet or amino-acid formulas. Some patients will tolerate progressive increase of enteral feeding making them able to be weaned from PN. However, normal growth velocity is not always achieved on full enteral feeding. Course may vary according to the concomitant liver disease, the severity of the digestive disease and the occurrence of infections. It is well reflected by the reported cases [Additional file 1]. It is important to note that the liver disease is, in some cases, already present at onset of diarrhea and before diagnosis is established. In these cases, the liver disease is due to the disease rather than to the PN, suggesting that the PN should be carefully adapted according to the liver disease. Indeed, the associated liver disease may be worsened by a long-term inappropriate PN (continuous PN, lipid free, complicated by infections *etc.*) with rapid course to end-stage liver disease and patient death.

Finally, the prognosis of this type of intractable diarrhea of infancy is poor, with >25% of the currently reported patients who died between the ages of 2 and 5 years, some of them with early onset of cirrhosis. In the largest but oldest series, five of the eight children reported died within 5 years due to sepsis or cirrhosis, despite aggressive intervention [9]. More recent case reports described better survival with long-term PN dependency or PN weaning in some cases [11-15]. In many patients, the severity of intestinal malabsorption and diarrhea makes them dependent on a daily long-term PN with subsequent risk of complications. However, it seems that some infants have milder phenotype with partial PN dependency or require only enteral feeding. In spite of adequate protein energy supplies, growth velocity remains low and final stature very short [9,11-14]. Attempts with recombinant human growth hormone was used in one patient (data unpublished), but failed to improve growth and final stature. Most of the reported patients have intellectual deficiency of variable severity. Some were too young when they died to be thoroughly evaluated.

## Conclusion

This very rare and heterogeneous syndrome has a poor prognosis with early death, liver cirrhosis or failure to thrive, and short stature with intellectual deficiency in the survivors. The heterogeneity of SD as well as the very different associated symptoms makes it very difficult to find an approach for understanding the genetic origin of the disease.

Prognosis has improved since the description of the first case with SD. Most patients now survive and about half of the patients may be weaned from PN at adolescence, but experience failure to thrive and final short stature.

## Supplementary Material

Additional file 1 Clinical features in patients with Syndromic (phenotypic) diarrhea. This table includes data from the published cases in the literature (patients 1–17, References 9–15). Patients 18 to 25 are currently under publication by the authors. Click here for file
