# Supplementary material for: Syndromic (phenotypic) diarrhea in early infancy
Source: Orphanet J Rare Dis. 2008 Feb 28;3:6. doi: 10.1186/1750-1172-3-6 (PMC2279108; doi:10.1186/1750-1172-3-6)
Supplement: Additional file 1 — Clinical features in patients with Syndromic (phenotypic) diarrhea. This table includes data from the published cases in the literature (patients 1–17, References 9–15). Patients 18 to 25 are currently under publication by the authors. [file 1750-1172-3-6-S1.doc]

| **PATIENTS** | **References** | **Consanguinity** | **Gestational age** | **BW**  **grs** | **Onset of Diarrhea (weeks)** | **Villous atrophy** | **Cirrhosis** | **Facial dysmorphy** | **Hair anomalies** | ***Tricorrhexis nodosa*** | **Mental retardation** | **Immune deficiency** | **Outcome** | **Follow up** | **Last**  **Status (*)** |
| --- | --- | --- | --- | --- | --- | --- | --- | --- | --- | --- | --- | --- | --- | --- | --- |
| 1 Siblings | Stankler et al  (10) | ND | 39 | 1680 | 2.5 | ? | + | + | + | + | Too young | + | Died | 33 days | _ |
| 2 Siblings | Stankler et al  (10) | ND | 34 | 1620 | 2 | ? | + | + | + | + | Too young | ? | Died | 87 days | - |
| 3 | Girault et al (9) | - | 34 | 2100 | 24 | Mild | - | + | + | + | no | + | Alive | 21 years | OF |
| 4 | Girault et al (9) | - | 37 | 1520 | 3 | Mild | - | + | + | + | + | + | Alive | 20 years | EN |
| 5 | Girault et al (9) | - | 37 | 1480 | 1 | severe | - | + | + | ? | - | + | Died | 38 months | - |
| 6 | Girault et al (9) | - | 40 | 1940 | 2 | severe | - | + | + | ? | + | + | Died | 26 months | - |
| 7 | Girault et al (9) | - | 40 | 2670 | 3 | severe | - | + | + | ? | - | + | Died | 20 months | - |
| 8 | Girault et al (9) | - | 35 | 1180 | 10 | severe | + | + | + | + | + | + | Died | 26 months | - |
| 9 | Girault et al (9) | + | 40 | 2000 | 6 | severe | + | + | + | + | + | + | Died | 58 months | - |
| 10 | Girault et al (9) | - | 39 | 1950 | 1.5 | Mild | - | + | + | + | + | + | Alive | 17 years | OF |
| 11  Siblings | Verloes et al (11) | - | 34 | 1410 | 2.5 | ? | + | + | + | - | Too young | + | Died | 6 mo | - |
| 12  Siblings | Verloes et al (11) | - | 37 | 1860 | 2 | Mild | + | + | + | - | Too young | ? | Died | ND | - |
| 13 | De vries et al (12) | - | Term | 1800 | 2 | + | no | + | + | + | + | + | Alive | ND | PN |
| 14 | Barabino et al (13) | - | 35 | 1345 | 5 | + | No | + | + | - | + | + | Alive | ND | PN |
| 15 | Landers et al (14) | ND | Term | ? | 2 | severe | + | + | + | + | + | + | Alive | 4 years | PN |
| 16 | Fabre et al (15) | - | 32 | 1240 | 5 | + | + | + | + | + | + | + | Alive | ND | PN |
| 17 | Fabre et al (15) | + | 40 | 2200 | 1 | - | + | + | + | + | + | + | Alive | ND | PN |
| 18 | Vinson et al (**) | - | Term | 2100 | 4 | Mild | No | + | + | + | + | + | Alive | 31 years | OF |
| 19 | Vinson et al (**) | - | 38 | 2140 | 3 | Mild | No | + | + | + | + | + | Alive | 13 years | PN |
| 20 | Vinson et al (**) | + | 40 | 1980 | 5 | severe | + | + | + | + | ++ | + | Died | 15 months | - |
| 21 | Vinson et al (**) | + | 35 | 1010 | 4 | Mild | + | + | + | + | no | + | Alive | 6 years | PN |
| 22 | Vinson et al (**) | - | 38 | 2860 | 3 | absent | No | + | + | + | Too young | + | Alive | 6 years | OF |
| 23 | Vinson et al (**) | - | 39 | 2000 | 1 | severe | No | + | + | + | + | + | Alive | 15 years | PN |
| 24 | Vinson et al (**) | - | 32 | 1240 | 5 | severe | No | + | + | + | + | + | Alive | 7 years | PN |
| 25 | Vinson et al (**) | + | 37 | 1045 | 1 | severe | + | + | + | + | ++ | + | Alive | 6 years | PN |

**(*) PN : parenteral nutrition ; EN : enteral nutrition ; OF : oral feeding**

**(**) Case currently on process of publication**
